# Robertsonian translocation between chromosomes (no.21/14) in relation to the history of spontaneous abortion in a family

**Published:** 2014-08

**Authors:** Mohammad Hasanzadeh-NazarAbadi, Fatemeh Baghbani, Iman Namazi, Salmeh Mirzaee

**Affiliations:** *Department of Medical Genetics, School of Medicine, Mashhad University of Medical Sciences, Mashhad, Iran.*

**Keywords:** *Spontaneous abortion*, *Balanced chromosomal rearrangement*, *Robertsonian translocation*, *Chromosomal abnormality*

## Abstract

**Background:** Approximately 205 million pregnancies occur each year in the worldwide. On the other hand, Spontaneous abortion has been reported in 15-20% of all diagnosed pregnancies. The most common cause of spontaneous abortion is chromosomal abnormalities of the embryo. Robertsonian translocation carriers specially 21-14 are the most common balanced rearrangement among the carrier couples with the history of spontaneous abortion. In order to search for balanced chromosomal rearrangement and cytogenetic disorders, 10 members of related family with consanguinity marriage with the history of recurrent miscarriage were assessed.

**Case: **Cytogenetic evaluation on the basis G-banding technique at high resolution was performed in 3 couples and their related family with the history of idiopathic RSA in order to postulate any balanced chromosomal rearrangement.

**Conclusion:** six members of them appeared with robertsonian balanced translocation between chromosome No.21 to No. 14 with the karyotype of 45, XX, t (14, 21) and 45, XY, t (14, 21), which this results are in agreement with several similar works which claimed that the risk of spontaneous abortion in couples with balanced chromosomal rearrangements is higher compared with general population. Considering to results of present study, it seems as if the cytogenetic analysis of couples with the history of recurrent abortions should be suggested compulsory to estimate the probable presence of any chromosomal rearrangement. This offer wills valuable information for genetic consulting.

## Introduction

Recurrent pregnancy loss (RPL) defined as two or more miscarriages before 20 weeks of gestation, affects up to 15-20% of couples (1, 2). It is proposed that the prevalence of chromosomal abnormalities in spontaneously aborted fetuses is as high as 50% which more than 80% of them occur within the first three months of gestation (3). Several factors are associated with such pregnancies complication, these are including: endocrinology imbalance, immune dysfunction, genetic disorder, advanced maternal age, gravity, body mass index (BMI) BMI >25, BMI <18, genital infectious, environmental toxins, anatomic uterine defects and chromosomal abnormalities (-). Although the cause of most habitual miscarriage is unknown nevertheless, parental chromosomal rearrangement is one the possible cause of such recurrent spontaneous abortion (RSA) in the first trimester of gestation (6). 

Centric fusion of two acrocentric chromosomes results in robertsonian translocation, which is one of the most common structural rearrangements in humans with an incidence of 1.23 per 1000 live birth. Carriers of robertsonian translocations are phenotypically normal, but they are at high risk of having chromosomally abnormal pregnancies (7). The evidence has been suggested that, the frequency of balanced rearrangement in couples with RSA is 20 fold higher than in the general population (8, 9). In the present study, 10 members of related family with consanguinity marriage (first cousin) with the history of recurrent miscarriage were assessed for cytogenetic disorders searching for balanced chromosomal rearrangement.

## Case report

A family with a history of RPL was referred to the Department of human genetics for cytogenetic evaluation. After obtaining informed consent, 3 ml of peripheral whole blood samples were drawn into sterile heparinized tube so coagulation prevented by additional of heparin. The mononuclear cells are cultured for three days in the presence of RPMI 1640 medium, supplemented with 20% of fetal calf serum, penicillin and streptomycin. The lymphocytes were stimulated by phytohemagglutinin to proliferate. At the end of this period, the culture was treated with colcemid, which disrupts mitotic spindles and prevents complication of mitosis. This greatly enriches the population of metaphase cells. The lymphocytes were harvested and treated briefly with hypotonic solution (0.05 M KCL). This makes the nuclei well osmotically. The swollen cells were fixed (with three part ethanol and one part acetic acid), dropped on to a microscope slide dried. Then, the cells were stained with trypsin-giemsa, in order to induce banding pattern.

Each chromosome was analyzed for its presence and pairing 46 human chromosomes in well-defined order, based on characteristic band. The 23 pairs differ in the length of the arms and each show unique banding pattern. 10 members of related family with consanguinity marriage (first cousin) with the history of recurrent abortion who were phenotypically normal were referred to medical genetics laboratory for cytogenetic analysis searching for balanced chromosomal rearrangement. 

**Plate 1:**
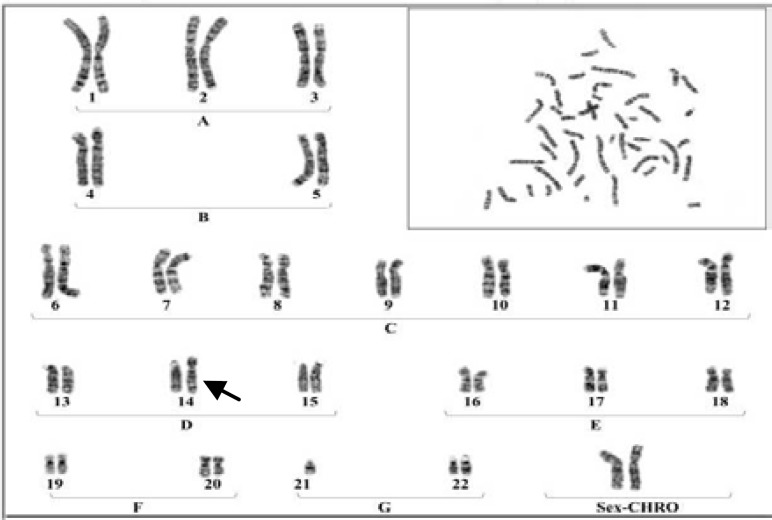
A Karyotype of the female with Robertsonian translocation between chromosomes No.21 to No 14

**Plate 2:**
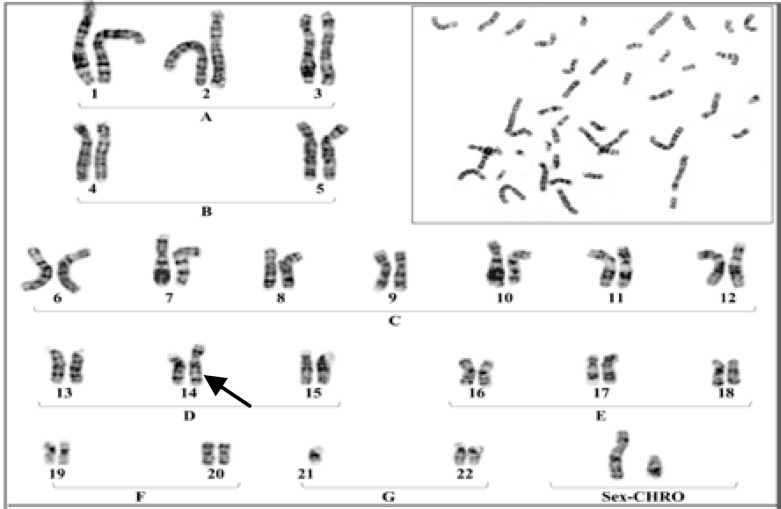
A Karyotype of a male with Robertsonian translocation between chromosomes No.21 to No 14

**Plate 3:**
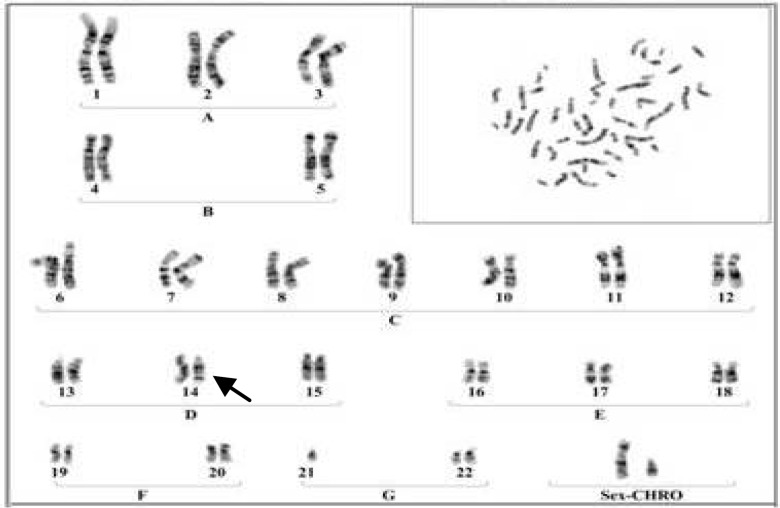
A Karyotype of a male with Robertsonian translocation between chromosomes No.21 to No 14

**Plate 4:**
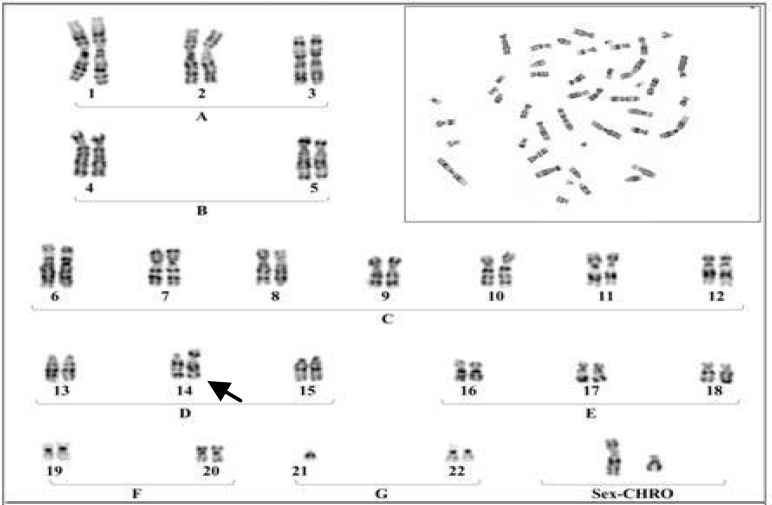
A Karyotype of a male with Robertsonian translocation between chromosomes No.21 to No 14

**Plate 5:**
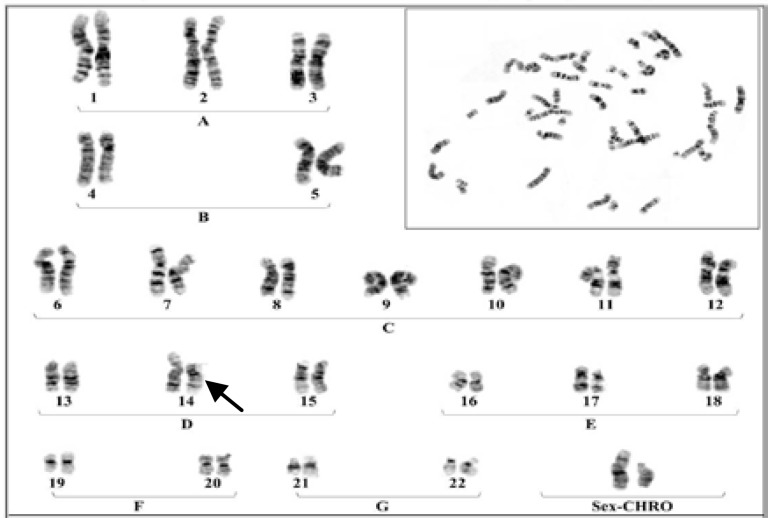
Proband's karyotype with down syndrome, 46, XY, t (21/14). (VI-3)

The results were indicated that in 6 members of these family appeared with similar balanced robertsonian translocation between chromosomes no.21/14 which 4 member and proband's Karyotype is represented in plate 1-5. Chromosomal constitution appeared with 21q/14q (q21.1, q11.2). 

**Figure 1:**
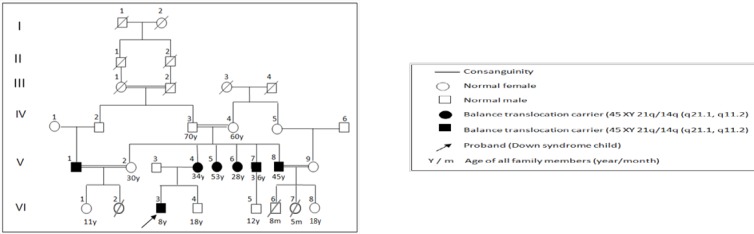
Pedigree of family with robertsonian translocation

45 XX 21q/14q (q21.1, q11.2). (V-4)

45 XY 21q/14q (q21.1, q11.2). (V-7)

45 XY 21q/14q (q21.1, q11.2). (V-8)

45 XY 21q/14q (q21.1, q11.2). (V-1)

## Discussion

Chromosomal rearrangement leads to reduced fertility in both gender. About 15-20% of pregnancies that end in spontaneous abortion occur in the first trimester. It has been reported that, the most common cause of spontaneous abortion in the first trimester (approximately 50%) is chromosomal abnormalities. The majority of chromosomal anomalies (95%) are numerical, about 60% are trisomy’s, 20% are X monosomy and the remainder are (15%) polyploidy especially triploidy (8). On the other hand, half of the structural abnormalities may be inherited from a parents who carrying a balanced chromosomal translocation which are at a higher risk of having children with chromosomal abnormalities (10, 11, 12). It has been reported in some related articles that, the risk of RSA is increased in couples who one of them has such balanced rearrangement (9, 10). 

Overall, carriers of balanced rearrangements are at high risk of recurrent spontaneous abortion, althought the phenotype of such couples seem to be normal, but neverthelese due to the production of unbalanced gametes, recurrent miscarriage or children with abnormal phenotypes might be observed (13, 14). In addition two recent studies show that a higher incidence of chromosomal translocation, directly increases the incidence of abortion in families while any increase in the incidence of chromosome inversion is not associated with an increased miscarriage in couples (13, 15). Cytogenetically, robertsonian translocation or centric fusion of two long arm of acrocentric chromosomes) involving chromosome 21 are the most common structural chromosomal aberrations, which occur with an incidence of ∼1 in 1000 in the general population (16). Although it seems that the prevalence of these structural abnormalities in males and females is similar, but a recent study found that women carrying Robertsonian translocation, carry the abnormality to the fetus four times more than men (16).

In our study 10 members of related family with the history of recurrent abortion were assessed for cytogenetic evaluation. The results were indicated that only 6 member of this family appeared with balanced Robertsonian translocation between chromosomes No.21/14 (plate 1-4). This results are in agreement with several previous studies which claimed that an increased risk of spontaneous abortion in couples with balanced chromosomal rearrangements compared with general population (2).

## Conclusion

Cytogenetic analysis of couples with the history of recurrent abortions should be suggested compulsory to estimate the probable presence of any chromosomal rearrangement. This offer will be valuable information for genetic consulting. 
